# Banded *vs* Bonded Space Maintainers: Finding Better Way Out

**DOI:** 10.5005/jp-journals-10005-1245

**Published:** 2014-08-29

**Authors:** Vikas Setia, Inder Kumar Pandit, Nikhil Srivastava, Neeraj Gugnani, Monika Gupta

**Affiliations:** Assistant Professor, Department of Pediatric Dentistry, Adesh Institute of Dental Sciences and Research, Bathinda, Punjab, India; Professor, Department of Pediatric Dentistry, D Av Dental College Yamuna Nagar, Haryana, India; Professor, Department of Pediatric Dentistry, Subharti Dental College Meerut, Uttar Pradesh, India; Professor, Department of Pediatric Dentistry, D Av Dental College Yamuna Nagar, Haryana, India; Professor, Department of Pediatric Dentistry, D Av Dental College Yamuna Nagar, Haryana, India

**Keywords:** Space maintainers, Prefabricated bands, Ribbond, Super splint, Survival rate

## Abstract

**Objectives:** Of this *in vivo* study was to evaluate various space maintainers in terms of survival rate, gingival health and presence of caries.

**Design:** A total of 60 extraction sites in the age group of 4 to 9 years were divided into four groups and different space maintainers were placed in them *viz* (conventional band and loop, prefabricated band with custom made loop, Ribbond, Super splint).

**Results:** Prefabricated bands with custom made loop showed maximum success rates (84.6%), while super splint (33.33%) was found to be least successful.

In terms of gingival health, prefabricated band with custom made loop reported minimum cases with poor gingival health (27.2%), while maximum cases with poor gingival health (50%) were reported with Super splint.

None of the space maintainers developed caries at the end of 9 months.

**How to cite this article:** Setia v, Pandit IK, Srivastava N, Gugnani N, Gupta M. Banded *vs* Bonded Space Maintainers: Finding Better Way Out. Int J Clin Pediatr Dent 2014;7(2):97-104.

## INTRODUCTION

Exfoliation of primary teeth and eruption of permanent teeth is a normal physiological process.^1^ When this normal process is disrupted, due to factors like premature loss of primary teeth, proximal carious lesions, etc. it may lead to mesial migration of teeth resulting in loss of arch length which may manifest as malocclusion in permanent dentition in the form of crowding, impaction of permanent teeth, supraeruption of opposing teeth, etc.^2^ The best way to avoid these problems is to preserve the primary teeth in the arch till their normal time of exfoliation.^3^ Hence, it is rightly quoted that primary teeth serve as best space maintainers for permanent dentition.

However, if premature extraction or loss of tooth is unavoidable due to extensive caries or other reasons, the safest option to maintain arch space is by placing a space maintainer.

The fixed space maintainers are usually indicated to maintain the space created by unilateral/bilateral premature loss of primary teeth in either of the arches. Of the various fixed space maintainers, band and loop type of space maintainers are one of the most frequently used appliances.^4^

Band and loop has been used since long as a space maintainer with high success rates^5-7^ but in spite of good patient compliance, disintegration of cement, solder failure, caries formation along the margins of the band and long construction time are some of the disadvantages associated with them.^3^

Considering this, there have been many pilot studies that explain the use of newer adhesive directly bonded splints, e.g. glass fiber reinforced composite resins (Ribbond,^8^ Everstick)^9,10^ as fixed space maintainers.

Ribbond is a biocompatible esthetic material made from high strength polyethylene fibers. The various advantages of this material include its ease of adhesion to dental contours, fast technique of application and good strength.^11^

Super splint is marketed as splinting material, consists of six layers of fine meshed very thin silanized glass fibers.^12^ It is compatible with all kinds of composite material and can be cut to necessary length. It is tear proof and easy to adapt.^12^ In a nutshell, its property to bond with tooth structure and silanized fibers getting polymerized with composite, makes it a viable choice for a space maintainer.

There are many options available to design various kinds of space maintainers. Each kind has some advantages over the other still there is lack of comparative studies in the literature comparing the efficacy of conventional banded type of band and loop with newly available bonded type fiber reinforced space maintainers.

Hence, the present *in vivo* study was planned to clinically evaluate various space maintainers in terms of survival rate, gingival health and presence/absence of caries.

## MATERIALS AND METHODS

Patients in the age range of 4 to 9 years visiting the Outpatient Department of Pedodontics and Preventive Dentistry, DAV (C) Dental College, Yamuna Nagar, were screened and the patients who either required extraction of the primary first/second molar or having pre-extracted primary 1st or 2nd molar in any of the arches were selected for the purpose of study. These patients were further screened on the basis of inclusion criteria.

Patients in the age group of 4 to 9 years, extraction sites with no space loss, erupting permanent tooth having adequate bone covering, fully erupted carious free teeth, patients with Dmf ≤ 4 were included in the study. Patients willing to participate in the study were selected, and informed consent was sought.

A brief history was recorded and clinical examination was done. Intraoral periapical radiographs were taken in areas of tooth loss. Study models were prepared, and space analysis was carried out for every child. for every selected child, oral prophylaxis was done prior to placement of space maintainer. Thirty-two patients were selected, and 60 extraction sites were selected for the study and called as samples. These were further divided into four groups (Table 1) and space maintainers were randomly placed in either the maxillary or mandibular arch having either single or multiple extraction sites.

**Table 1:** **Table 1:** Division of samples

*Groups*		*Space maintainer used*		*Sample size*	
I		Conventional band and loop		15	
II		Prefabricated band with custom made loop		15	
III		Ribbond		15	
IV		Super splint		15	

### Technique of Band Fabrication (Group I)

**Figs 1A and B:**
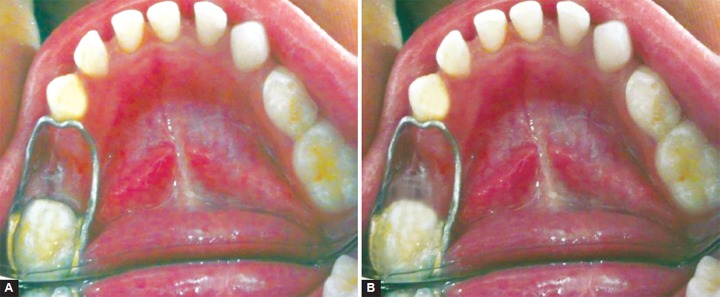
(A) Immediate postoperative conventional band and loop and (B) 9 months

A conventional band and loop was fabricated according to the technique described by finn,^13^ and patient was instructed not to eat or drink for 30 minutes and not to bite any hard food. The patient was recalled after 3 months (Figs 1A and B).

### Technique of Application of Prefabricated Bands (Group II)

**Figs 2A and B:**
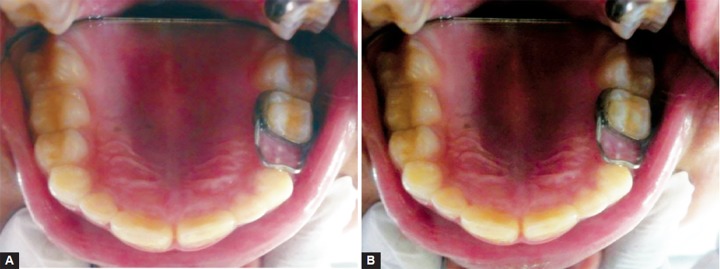
(A) Immediate postoperative prefabricated band with custom made loop and (B) 9 months

A prefabricated band was selected for the abutment tooth by measuring the mesiodistal diameter of abutment tooth with a caliper and correlating it with the internal diameter of the prefabricated band. It was ensured that it covered the entire surface of tooth. The loop was then soldered with the band in its middle third. Patient was instructed not to eat or drink for 30 minutes and not to bite any hard food. The patient was recalled after 3 months (Figs 2A and B).

### Technique of Application of Glass Fiber Reinforced Composite Resins (Groups III and IV)

**Figs 3A and B:**
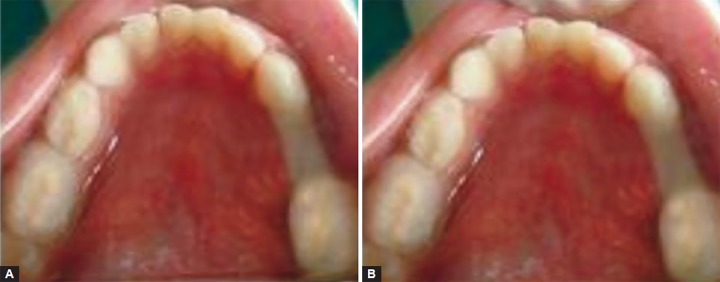
(A) Immediate postoperative Ribbond and (B) 9 months

The amount of Ribbond to be placed was measured with Vernier caliper. The abutments were cleaned with pumice, isolated, acid etched with 37% orthophosphoric acid, washed with water and then dried. The bonding agent (3M) was applied and cured for 10 seconds according to manufacturers instructions. A thin layer of fowable composite (Flow Line, Heraeus Kulzer, Germany) was applied on distal surface of the mesial tooth, and on the mesial surface of the distal tooth, and Ribbond was placed between these abutment teeth as a saddle. After preliminary curing, at each end of fiber network for 40 seconds additional restorative composite was further placed over the area where fiber contacted the tooth and light cured for 40 seconds to completely bond the space maintainer with the abutment (Figs 3A and B).

The space maintainer was checked for any occlusal and gingival interference. finishing was done with composite finishing burs. Patient was instructed not to bite any hard foods.

**Figs 4A and B:**
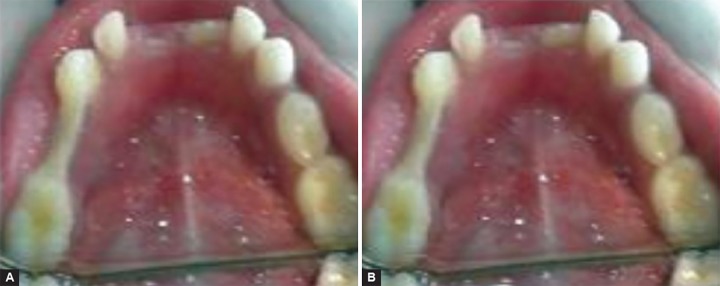
(A) Immediate postoperative Super splint and (B) 9 months

Super splint (Figs 4A and B) was applied in the same manner as in group III (Ribbond).

Instructions for oral hygiene and appliance maintenance were given to children of their parents. Patients were recalled at 3, 6 and 9 months interval for evaluation of space maintainers using the following criteria (Table 2):

Clinical evaluation of the patient was carried out by both visual and tactile examination to check for the survival rate, presence of caries and gingival health at 3rd, 6th and 9th month recall, according to the following criteria:

### Survival Rate

The survival rate was checked as per following comparable criteria:^14^

- Lost to follow-up (LF)
- Failed (F)
- Successful (S)
- Censored at the end of study (C).

### Caries

The presence of dental caries was checked visually and with a explorer at 3, 6, 9 months according to the following scale:

- Presence of caries – ‘P’
- Absence of caries – ‘A’

### Gingival Health

Plaque deposition of the abutment tooth of the space maintainer was evaluated according to the index used by Sillness and Loe H.^15^

The results obtained from the above-mentioned criteria were tabulated accordingly and evaluated for statistical significance.

The data collected at 3, 6 and 9 months interval was then tabulated and statistically analyzed using the Chi-square test and McNemar’s test.

## OBSERVATIONS AND RESULTS

The data obtained was statistically analyzed by the statistical software SPSS version 13.0. There were 15 subjects in each of four groups at the time of recruitment and each subject was followed up at three time points, i.e. 3, 6 and 9 months. Since, it is a follow-up study each group had some ‘lost to follow-up’ subjects. Group I had 100% follow-up, group II had 13 out of 15 (86.7%), group III had 11 out 15 (73.3%) and group IV had 12 out of 15 (80.0%) follow-up at 3 months. There was no follow-up loss at 6 and 9 months. The analysis was done on the available subjects assuming that within a given group the subject follow same proportion of outcome variables, e.g. in a group, 1 out 13 subjects fail, that means 7.7% subjects failed at 3 months and it was assumed that same failure rate will be in lost to follow-up subjects. If the failure had occurred there, we would have excluded those subject for further evaluation for gingival health and caries.

The data obtained was statistically analyzed using Chisquare test. Chi-square test is an important non-parametric test. We require only the degree of freedom for using this test.

Comparison at the given point of time within the group was done using McNemar’s test. McNemar’s test is used because we are comparing the same subjects, in other words, data are correlated. p-value less than 0.05 was considered as significant.

**Table 2:** **Table 2:** Criteria evaluated

		*Inspection method*		*Rating*	
Survival rate		Visual inspection		Successful Failed Lost to follow-up Censored at the end of study	
Caries		Visual inspection with explorer and mirror		Absent Present	
Plaque		Visual inspection with explorer and mirror		Good Fair Poor	

**Graph 1:**
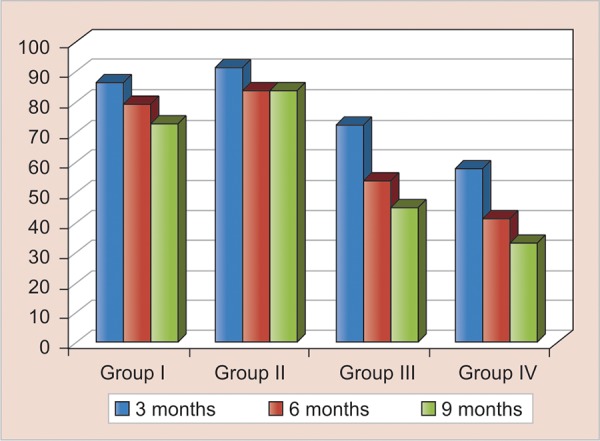
Success rates for different groups at 3, 6 and 9 months

Prefabricated band with custom made loop showed the maximum success rate at the end of 9 months. Significant difference was observed in the success rates of prefabricated band with custom made loop in comparison to the other groups (Table 3 and Graph 1).

**Graph 2:**
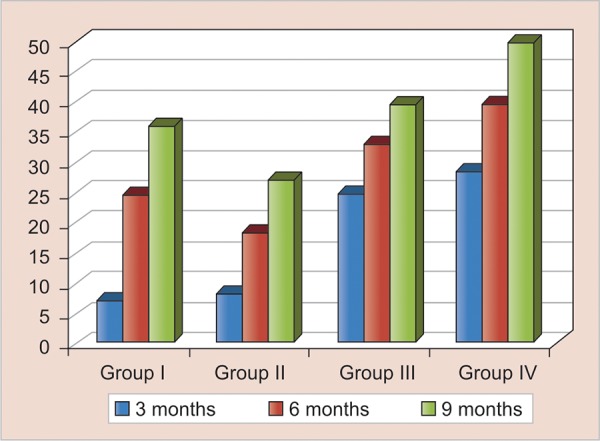
Percentage of poor gingival health for different groups

Gingival health (plaque) was observed at 3, 6 and 9 months. Super splint reported the poorest gingival health at all recall periods. However, prefabricated band with custom made loop reported with best gingival health among the four groups but the results were not statistically significant at all the recall appointments (Table 4 and Graph 2).

None of the patients developed caries at the end of study period in all the four groups.

## DISCUSSION

Conventional band and loop has long been used for maintaining space in cases of premature single tooth loss.^4^ However, it has the following disadvantages *viz* tendency for disintegration of cement, inability to prevent rotation or tipping of adjacent teeth, increased chairside and laboratory time.^16^ The prefabricated bands were introduced in November 1935^17^ since pinching of the band required expensive, time consuming and customized procedures, the prefabricated bands evolved for saving time. They are available in a variety of sizes to fit molar teeth.

However, the limitations of conventional prefabricated band and loop remain the same. This indicates the need for newer designs and materials of appliance. One such material is glass fiber-reinforced composite resins (FRCRs) which are available to the pediatric dental market and it can be used as an alternative to the conventional space maintainer.^8^

Fiber-reinforced composite resins have been used in removable prosthodontics, fixed partial dentures, periodontal splints and in orthodontic treatment as a retention splint.

**Table 3:** **Table 3:** Comparison of survival rate among different groups

*Evaluation period*		*Conventional band and loop (n = 15)*		*Prefabricated band with custom made loop (n = 13)*		*Woven polyethylene fibers ribbond (n = 11)*		*Super splint (n = 12)*		*p-value (Chi-square)*			
3 months		13 (86.7%)		12 (92.3%)		8 (72.7%)		7 (58.3%)		0.172		NS	
6 months		12 (80.0%)		11 (84.6%)		6 (54.5%)		5 (41.7%)		0.068		NS	
9 months		11 (73.3%)		11 (84.6%)		5 (45.5%)		4 (33.3%)		0.029		S	

NS: Nonsignificant; S: Significant

**Table 4:** **Table 4:** Comparison of gingival health among different groups

*Evaluation period*		*Conventional band and loop (n = 15)*		*Prefabricated band with custom made loop (n = 13)*		*Ribbond (n = 11)*		*Super splint (n = 12)*		*Exact significane*		*Inference*	
No. of subjects available for gingival health at 3 months		13		12		8		7		–		–	
Three months poor		1 (7.6%)		1 (8.3%)		2 (25%)		2 (28.6%)		0.456		NS	
No. of subjects available for gingival health at 6 months		12		11		6		5		–		–	
Six months poor		3 (25%)		2 (18.8%)		2 (33.3%)		2 (40%)		0.779		NS	
No. of subjects available for gingival health at 9 months		11		11		5		4		–		–	
Nine months poor		4 (36.3%)		3 (27.2%)		2 (40%)		2 (50%)		0.949		NS	

NS: Nonsignificant

Ribbond and super splint provide an excellent esthetic choice as a space maintainer. However, there are limited reports to compare the various designs of space maintainer in terms of efficacy and longevity. Hence, the present study was undertaken to clinically evaluate various space maintainer with regard to their survival rate, gingival health and presence/absence of caries at the difference of 3, 6 and 9 months time interval.

The patients who either required extraction of the primary first/second molar or having pre-extracted primary 1st or 2nd molar in any of the arches were selected for the purpose of study. These patients having single or multiple extraction sites, were indicated for the use of space maintainers. These were further divided into four groups, and space maintainers were randomly placed in the maxillary or mandibular arch having either single or multiple extraction sites.

## SURVIVAL RATE

Prefabricated band and loop reported maximum success rate at 9th month (84.6%) whereas minimum success rate was reported with super splint was 33.33%. Conventional band and loop and Ribbond had success rate of 73.3 and 45.4% respectively.

In the present study, it was observed that the success rate at the third month recall was 86.6% in conventional band and loop (group 1). The success rate at 6th and 9th months were 80 and 73.3% respectively.

Though extreme care was exercised to follow the ideal steps for band formation, fabrication of space maintainer appliance and its cementation, still there might have been some failure at the band cement interface, leading to failure of space maintainer.

Space maintainer might have also failed because patient might not have followed the postoperative instructions.

Our results were in accordance with the study conducted by Qudeimat mA et al^18^ and by Baroni C et al.^5^ In our study, also most of the appliances failed due to cement loss.

For prefabricated band with custom made loop (group II), 92.3% of the cases reported success at 3rd month recall; while, at the 6th and 9th months recall, success rate was 84.6%. Preformed bands have the advantage of not requiring cold working usually required for fabrication of custom made band. moreover, they are usually available in varying sizes to appropriately fit occlusogingival height of abutment tooth and are known to fit the contours of the tooth.

Still the chances of failure of a space maintainer made of prefabricated band can be due to the failure of ‘band-cementtooth’ interface or breaking of soldering joint between loop and the band.

Most of the failures seen with prefabricated band with custom made loop was due to failure at the tooth cement interface.

The results of our study were in accordance to studies conducted by Rajab DL,^19^ Moore TR, Kennedy DB (2006) who observed that the most common cause of failure of space maintainers was cement loss.

The success rate of prefabricated band with custom made loop at the end of study period was higher than group I (84.6%). There are no clinical studies comparing the retention of conventional band with prefabricated band but the good results obtained may be attributed to band being prefestooned and presence of buccal groove.

For Ribbond (group III), 72.9% of cases reported with success at 3rd month and, at 6th month, the success rate was 54.5%, similarly the success rate at 9th month was 45.4%.

Ribbond is only bonded to natural tooth in form of a bridge, with its saddle bonded to both teeth (mesial and distal to edentulous space and this saddle is actually maintaining the required space for permanent successor. Thus, the hanging fiber bridge is subjected to compressive and tangential forces that might lead to fracture of fiber frame. Secondly, the transmission of forces from fiber frame to bonding margins between tooth and Ribbond on either side of framework might have weakened the bond and would have caused debonding of fiber composite interface or enamel cement interface.

In our study, we reported failure at the enamel composite interface this might be mainly due to irregular tangential acting on the fiber bridge.

Our results are in accordance with Kirzioglu Z^3^ as they also found the main cause of failure was debonding at the enamel composite interface.

Kargul B et al^9^ and Subramanium P^20^ reported similar success rates as compared to our study with glass FRCRs as space maintainer but our results were contrary to those described by Kirzioglu Z^3^ who reported failure rates of (67-94%).

Super splint (group IV) group reported the minimum success rate with 58.3% at 3rd month, 41.7% at 6th month and 33.33% at the 9th month. Super splint is a silanized fiber^17^ similar to Everstick^8^ and Splint In.^3^

It is a material indicated for splinting but being a similar kind of silinated fiber it makes all the sense to give it a trial to use it as a space maintainer. As far as our knowledge, we could not find any study in the literature justifying its percentage of failure. But the failure might have occurred due to overzealous finishing of the composite resins, lack of contact in the edentulous areas.

No statistically significant differences were found between all groups at 6th and 9th month recall. Statistically, significant differences (p < 0.05) were noted between all the groups at the 9th month recall. Intergroup comparison was done at 9th month recall. The results revealed statistically significant difference (p < 0.05) between group IV (Super splint) and group II (prefabricated band with custom made loop). This might be due to the fact that the method of cementation of appliance in group II is different from tangential forces acting on the space maintainer in group IV.

Group II showed the maximum success rate followed by groups I, III and then group IV.

### Presence/Absence of Caries

The caries was examined visually and by tactile method. An explorer was used to check for the presence of caries on the abutment tooth in the patients.

None of the patients developed caries throughout our study over the time period of 9 months.

Space maintainer was cemented in groups I and II using luting GIC. After insertion of the appliance, the patients were thoroughly instructed about proper guidance for maintenance of oral hygiene and educated about proper brushing techniques. In our study, good oral hygiene and fluoride releasing capacity of GIC might have attributed to no caries development in groups I and II.

Our results were in accordance with the study conducted by P Subramaniam, Basu GK, Sunny R,^20^ Karman L,^8^ Kargul B.^9^

In contrast, prevalence of enamel opacities increased after orthodontic treatment in a cross-sectional study by Mizhari E et al^21^ but these enamel opacities were observed after debanding of appliance and their location was underneath the band and not at the margins of the band and tooth. Thus, explaining the contraindications of the results.

Gorelick L et al^22^ also in their study reported increased incidence of white spot lesion formation after banding which was again contraindicating to our results but the difference have been attributed to the fact that they evaluated the incidence of white spot lesions in multibanded appliances, which might have caused hindrance to proper maintainence of oral hygiene that might have led to the presence of white spots.

Groups III and IV did not develop any caries. We could not find any studies to report our findings. We hypothesized that zero caries incidence might be due to complete fushing of the material with the abutment tooth and maintenance of good oral hygiene with no additional food retentive areas and consequently no development of caries.

### Gingival Health

Regarding gingival health nonsignificant differences were obtained between the four groups of space maintainers at 3rd, 6th and 9th months recall (p < 0.05).

Prefabricated band and loop also reported minimum cases of poor gingival health at 9th month (27.2%) and Super splint (50%) again reported maximum cases of poor gingival health.

Health of gingiva is inversely proportional to the presence of plaque. Any kind of appliance that would increase plaque formation or lodgement would hamper gingival health.

In group I at 3 months recall, only 17.6% subject had poor gingival health. Similarly at 6 months recall, 25% of patients had poor gingival health. The patients with poor gingival health at the end of 9 months were 36.4%.

In group II, gingival health at the 3rd month recall 8.3% of patients had poor gingival health, at 6 months recall, 18.18% subject had poor gingival health. At the 9 months recall, 27.2% had poor gingival health.

In groups I and II, space maintainers were fabricated using custom made and prefabricated band along with a loop respectively. Such metallic bands can easily cause plaque traps to form. Though in our study, we took all precautions to form smoothest solder margins with no undercuts and patients were also instructed to maintain good oral hygiene but, in spite of all these precautions, some plaque retentive traps might have formed leading to poor to fair gingival health among different patients.

Studies show that the plaque index (Pl) increased significantly on banded teeth as compared with control sites.^23^

Group II had better gingival health of the abutment tooth than group I, this might be due to the better adaptation of bands to the tooth, hence, formation of less plaque retentive areas.

In conclusion, it can be said that prefabricated band with custom made loop may be a viable alternative to conventional band and loop since it has somewhat more success rate and less plaque deposition.

For group III at 3 months recall, 25% patients had poor gingival health; at 6 months recall, 33.33% patients had poor gingival health and, at 9 months recall, 40.0% of patients had poor gingival health with 60.0% having fair gingival health.

In group IV at 3rd month recall, 28.57% patients had poor gingival health. At 6th month recall, 40% patients had poor gingival health, while at 9 months recall, 50.0% of patients had poor gingival health.

Groups III and IV observed higher proportions of patients with poor gingival health as compared to groups I and II, this might be attributed to plaque retentive sites along the fiber framework.

Moreover, there might have been Hawthorne effect among groups I and II patients leading to more frequency of brushing and in turn fair gingival health.

Group IV patients observed high percentage of poor gingival health. This might may also be due to the fact that many of the patients ‘lost to follow-up’ over the period of study. Other possible reason might be that, the strength of this material was less as compared to group III which might have led to formation of a ‘gap’ at the enamel composite interface.

## CONCLUSION

Prefabricated band with custom made loop exhibited maximum success rate followed by conventional band and loop and then Ribbond. Minimum success rate was shown by Super splint.

Prefabricated band and loop showed better gingival health followed by conventional band and loop and then Ribbond. Super splint observed poorest gingival health among all the four experimental groups.

No incidence of caries was observed in all the four experimental groups till the end of the study.

Woven polyethylene fibers (Ribbond) and polysilane fibers (Super splint) have a number of advantages *viz* no lab procedure is required, better esthetic and better tolerance by the patient but, since their success rates were found to be low, they may be recommended for short period of time. Banded space maintainers still remain the gold standard in the management of space in pediatric dental practice. Hoever, long-term clinical trials are needed to evaluate their results.
